# Neurological Consequences of SARS-CoV-2 Infection and Concurrence of Treatment-Induced Neuropsychiatric Adverse Events in COVID-19 Patients: Navigating the Uncharted

**DOI:** 10.3389/fmolb.2021.627723

**Published:** 2021-02-18

**Authors:** Pobitra Borah, Pran Kishore Deb, Balakumar Chandrasekaran, Manoj Goyal, Monika Bansal, Snawar Hussain, Pottathil Shinu, Katharigatta N. Venugopala, Nizar A. Al-Shar’i, Satyendra Deka, Vinayak Singh

**Affiliations:** ^1^ School of Pharmacy, Graphic Era Hill University, Dehradun, India; ^2^ Department of Pharmaceutical Sciences, Faculty of Pharmacy, Philadelphia University, Amman, Jordan; ^3^ Department of Anesthesia Technology, College of Applied Medical Sciences in Jubail, Imam Abdulrahman bin Faisal University, Dammam, Saudi Arabia; ^4^ Department of Neuroscience Technology College of Applied Medical Sciences in Jubail, Imam Abdulrahman bin Faisal University, Dammam, Saudi Arabia; ^5^ Department of Biomedical Sciences, College of Clinical Pharmacy, King Faisal University, Al-Ahsa, Saudi Arabia; ^6^ Department of Pharmaceutical Sciences, College of Clinical Pharmacy, King Faisal University, Al-Ahsa, Kingdom of Saudi Arabia; ^7^ Department of Biotechnology and Food Technology, Durban University of Technology, Durban, South Africa; ^8^ Department of Medicinal Chemistry and Pharmacognosy, Faculty of Pharmacy, Jordan University of Science and Technology, Irbid, Jordan; ^9^ Pratiksha Institute of Pharmaceutical Sciences, Chandrapur Road, Panikhaiti, Guwahati, India; ^10^ Drug Discovery and Development Centre (H3D), University of Cape Town, Rondebosch, South Africa; ^11^ South African Medical Research Council Drug Discovery and Development Research Unit, Department of Chemistry and Institute of Infectious Disease and Molecular Medicine, University of Cape Town, Rondebosch, South Africa

**Keywords:** COVID - 19, SARS – CoV – 2, neurotropism, nervous system, neurologic manifestations, psychological impact, neuropsychiatric adverse effects, drug-drug interaction

## Abstract

Severe acute respiratory syndrome coronavirus 2 (SARS-CoV-2) binds to the angiotensin-converting enzyme 2 (ACE2) receptor and invade the human cells to cause COVID-19-related pneumonia. Despite an emphasis on respiratory complications, the evidence of neurological manifestations of SARS-CoV-2 infection is rapidly growing, which is substantially contributing to morbidity and mortality. The neurological disorders associated with COVID-19 may have several pathophysiological underpinnings, which are yet to be explored. Hypothetically, SARS-CoV-2 may affect the central nervous system (CNS) either by direct mechanisms like neuronal retrograde dissemination and hematogenous dissemination, or via indirect pathways. CNS complications associated with COVID-19 include encephalitis, acute necrotizing encephalopathy, diffuse leukoencephalopathy, stroke (both ischemic and hemorrhagic), venous sinus thrombosis, meningitis, and neuroleptic malignant syndrome. These may result from different mechanisms, including direct virus infection of the CNS, virus-induced hyper-inflammatory states, and post-infection immune responses. On the other hand, the Guillain-Barre syndrome, hyposmia, hypogeusia, and myopathy are the outcomes of peripheral nervous system injury. Although the therapeutic potential of certain repurposed drugs has led to their off-label use against COVID-19, such as anti-retroviral drugs (remdesivir, favipiravir, and lopinavir-ritonavir combination), biologics (tocilizumab), antibiotics (azithromycin), antiparasitics (chloroquine and hydroxychloroquine), and corticosteroids (dexamethasone), unfortunately, the associated clinical neuropsychiatric adverse events remains a critical issue. Therefore, COVID-19 represents a major threat to the field of neuropsychiatry, as both the virus and the potential therapies may induce neurologic as well as psychiatric disorders. Notably, potential COVID-19 medications may also interact with the medications of pre-existing neuropsychiatric diseases, thereby further complicating the condition. From this perspective, this review will discuss the possible neurological manifestations and sequelae of SARS-CoV-2 infection with emphasis on the probable underlying neurotropic mechanisms. Additionally, we will highlight the concurrence of COVID-19 treatment-associated neuropsychiatric events and possible clinically relevant drug interactions, to provide a useful framework and help researchers, especially the neurologists in understanding the neurologic facets of the ongoing pandemic to control the morbidity and mortality.

## Introduction

On January 30, 2020, the WHO declared the Coronavirus Disease-2019 (COVID-19) outbreak a Public Health Emergency of International Concern (WHO, 2020b). The genome sequencing analysis has confirmed that the causative novel coronavirus (CoV) belongs to the lineage B of beta-coronavirus, and is named as Severe Acute Respiratory Syndrome Coronavirus 2 (SARS-CoV-2) by the International Committee on Taxonomy of Viruses. It exhibited 96.2, 79.5, and 50% similarity in gene sequence with the earlier known bat CoV RaTG13, SARS-CoV, and Middle East Respiratory Syndrome Coronavirus (MERS-CoV), respectively (Jin Y. et al., 2020). Thus, the probable natural host of the virus is suspected to be the bats, possibly transmitted to humans through an unknown intermediate (Guo et al., 2020). The general clinical manifestations of SARS-CoV-2 infection may range from the onset of self-limiting signs and symptoms related to upper respiratory tract infection like rhinorrhea and sore throat to nonspecific clinical conditions such as non-productive cough, fever, dyspnoea, and difficulty in breathing (Kang and Xu, 2020). Although SARS-CoV-2 predominantly affects the respiratory system, recent evidences suggest the neurological involvement with COVID-19 (Asadi-Pooya and Simani, 2020; Helms et al., 2020a; Mao et al., 2020). However, neurotropism and neuroinvasive mechanism of SARS-CoV-2 is still under debate. The expression of angiotensin-converting enzyme 2 (ACE2) receptor, required for cell tropism, has recently been demonstrated to be present on neurons and glial cells of different brain regions (Palasca et al., 2018; Muus et al., 2020), including the striatum, cerebral cortex, posterior hypothalamic area, substantia nigra, and the brain stem (Chen R. et al., 2021). Several hypotheses suggest the possibility of SARS-CoV-2 transmission into the central nervous system (CNS) via the hematogenous pathway, olfactory bulb invasion, and retrograde axonal transport (Desforges et al., 2020). Notably, not all neurological manifestations involve direct neuroinvasive mechanisms. For instance, indirect neurologic symptoms may result from the exacerbated systemic pro-inflammatory responses (Zhou et al., 2020). In addition to that, post-infection neurological complications and psychological issues associated with COVID-19 are also of great concern.

The therapeutic potential of certain repurposed drugs has led to their off-label use against COVID-19, such as anti-retroviral drugs (remdesivir, favipiravir, and lopinavir-ritonavir combination), biologics (tocilizumab), antibiotics (azithromycin), antiparasitics (chloroquine and hydroxychloroquine), and corticosteroids (dexamethasone) (Borah et al., 2020). On top of the neurological complications associated with SARS-CoV-2 infection, many of these drugs potentially exhibit certain clinical neuropsychiatric adverse events. For example, previous studies have reported the chloroquine-/hydroxychloroquine-associated neuropsychiatric adverse effects such as seizures, ataxia, retinopathy, and limbic encephalitis (Maxwell et al., 2015). Similarly, corticosteroids can provoke agitation, anxiety, depression, delusion, and hallucinations (Ou et al., 2018). Both corticosteroids and anti-viral drugs may also trigger convulsions and seizures (van Campen et al., 2018; Jarrahi et al., 2020). Apart from these, the potential COVID-19 therapies may also interact with the prescribed medications for pre- and/or co-existing neuropsychiatric diseases demonstrating serious drug-drug interactions, thereby, further complicating the clinical outcomes. From this perspective, this review will discuss the possible neurological manifestations and sequelae of SARS-CoV-2 infection with emphasis on the probable underlying neurotrophic as well as neuroinvasive mechanisms. Additionally, we will highlight the concurrence of COVID-19 treatment-associated neuropsychiatric events and possible clinically relevant drug interactions, to provide a useful framework and help researchers, especially the neurologists in understanding the neurologic facets of the ongoing pandemic to control the morbidity and mortality.

## SARS-CoV-2 Pathophysiology

SARS-CoV-2 are non-segmented positive single-stranded RNA containing spherical shaped viruses with crown-like lipid envelope (Hasöksüz et al., 2020; Lundstrom, 2020). The basic genome of the virus comprises of orderly arranged 5' methylated (UTR) caps, open reading frames (ORFs), spike (S), envelope (E), membrane (M), nucleocapsid (N), and other accessory proteins (Malik, 2020)**.** The S proteins are membrane fusion proteins (type I) associated with receptor-binding function mediated through the receptor binding-domains (RBD) and assist in the virus fusion to the host cell membrane. On the other hand, E proteins contribute to the virion assembly and their release, and M proteins characterize the viral envelope shape, while N proteins package the viral genome to form the complete virion (Alanagreh et al., 2020; Malik, 2020). Several other accessory proteins present in the genome are known to possess overlapping compensatory roles.

**FIGURE 1:**
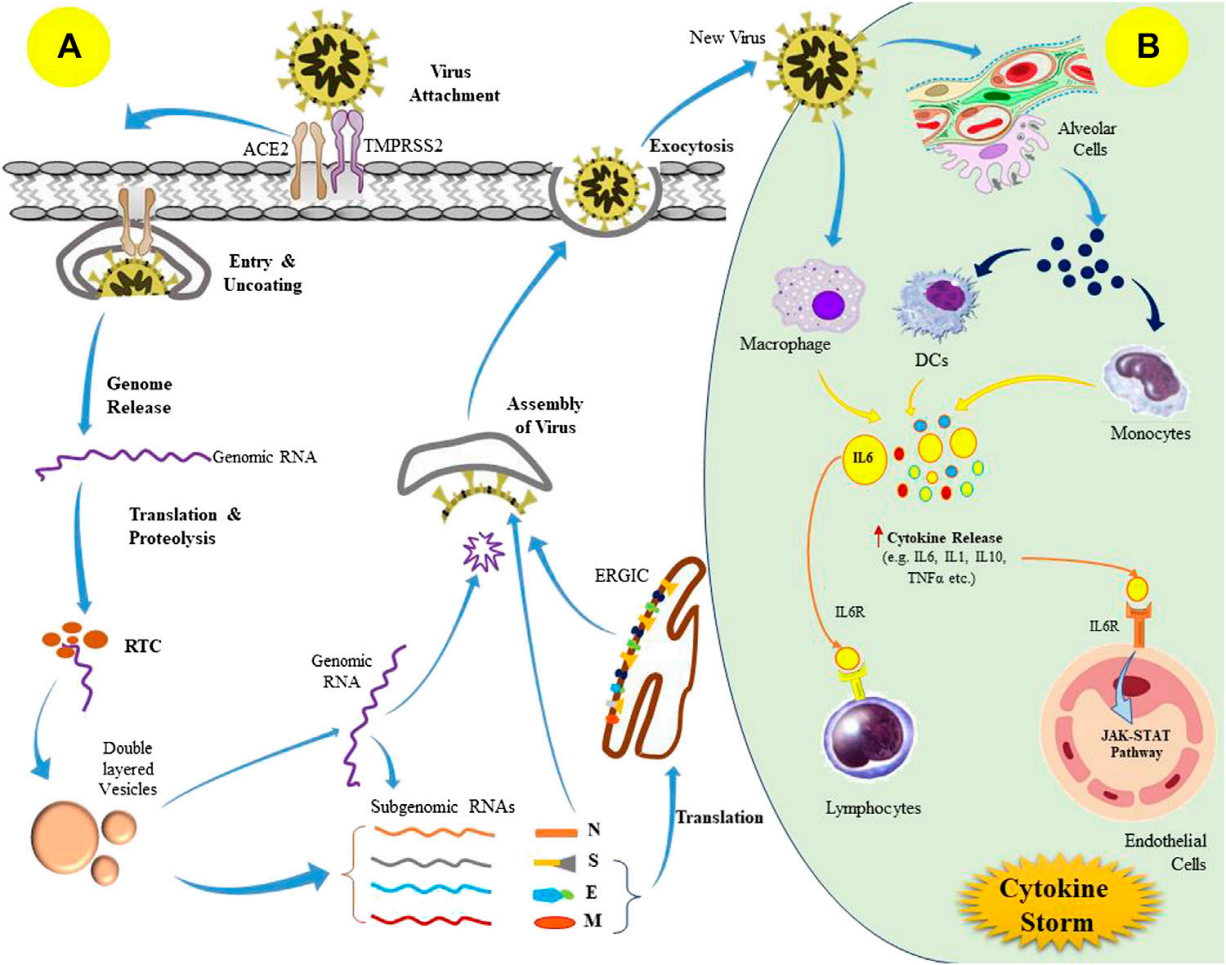
Pathogenesis of SARS-CoV-2 (severe acute respiratory syndrome coronavirus 2) infection; **(A)** Replication cycle of SARS-CoV-2 virus, and **(B)** Induction of cytokine storm in the host following viral infection.

The replication cycle of the SARS-CoV-2 begins once the virus transmits into the host body via interaction of S proteins with the ACE2 receptor of the target cells such as type II alveolar cells, tracheobronchial epithelial, vascular endothelial, and the macrophages (Hamming et al., 2004; Kotta et al., 2020). The S protein is further cleaved by the acid-dependent proteolysis with various proteases including transmembrane protease serine 2 (TMPRSS2) and cathepsin, which leads to membrane fusion and viral genome released into the host cell cytoplasm (Belouzard et al., 2009). The released SARS-CoV-2 polyproteins are processed by the major proteases such as papain-like protease (PLpro) and 3-chymotrypsin-like protease (3CLpro) to synthesize several non-structural proteins (NSPs) like RNA-dependent RNA polymerase (RdRP). Eventually, it leads to the formation of a double-layered vesicle with a replication-transcription complex (RTC) that produces sub-genomic RNAs, acting as templates for the translation of structural as well as other accessory proteins (Ahn et al., 2020). Later, the S, E, and M proteins (formed via translation) enter into the endoplasmic reticulum-Golgi intermediate compartment (ERGIC) and undergo viral assembly along with N protein and genomic RNA. Ultimately, it produces mature virions inside the vesicles, which are released from the cell by the process of exocytosis (Figure 1) (Malik, 2020).

These newly released viruses can provoke cellular injury as well as pyroptotic death in the infected host cells during the invasion and replication process, which ultimately leads to vascular leakage of the viral components (Tay et al., 2020; Yang, 2020). The recognition of the released viral components by macrophages and alveolar epithelial cells via pattern recognition receptors triggers the production of excess pro-inflammatory cytokines, including interleukin-6 (IL-6), monocyte chemo-attractant protein 1 (MCP1), interferon γ (IFNγ), and IFNγ-induced protein 10 (IP-10), which causes a local wave of inflammation (Huang et al., 2020). This cytokine-mediated inflammatory signaling leads to pulmonary infiltration by the T-lymphocytes and monocytes (Tian et al., 2020). Usually, these immune cell recruitment aims to eradicate the pulmonary infection, but in some cases, there may be an impaired immunological response that initiates an anomalous inflammatory phenomenon referred to as “cytokine storm” (Figure 1) (Mehta et al., 2020; Ye et al., 2020). This may further lead to cytokine release syndrome (CRS)**.** Furthermore, unrestricted infiltration also facilitates inflammatory injury to the pulmonary tissues due to the excess discharge of proteases and reactive oxygen species (ROS). Overall, it prompts diffuse alveolar damage as well as exudative pulmonary edema that results in acute respiratory distress syndrome (ARDS) characterized by inefficient pulmonary gas exchange, severe breathing complications, and a drop in blood oxygen saturation (Tian et al., 2020). Notably, cytokine storm is not only restricted to local damage, instead it has a rippling effect throughout the organ systems, and leads to septic shock and multi-organ failure (Ruan et al., 2020).

Usually, the incubation period of the SARS-CoV-2 ranges from 5 to 6 days (maximum 14 days) (WHO, 2020a), and the clinical manifestations include the onset of sign and symptom associated with upper respiratory tract infection like rhinorrhea and sore throat, followed by non-productive cough, headache, dizziness, hyposmia, hypogeusia fever, myalgia, dyspnoea, fatigability, confusion, and confirmed pulmonary lesions as observed on chest radiography (Deb et al., 2020; Kang and Xu, 2020). Moreover, pre-existing conditions like diabetes, hypertension, chronic respiratory disease, chronic liver diseases, obesity, chronic kidney diseases, cancers, and other cerebrovascular diseases have shown a significant correlation with disease progression and severity (Guan et al., 2020; NIH, 2020). The next section will emphasize the neuropsychiatric manifestations that have been reported till now. As surveillance data is still evolving, various reports and case series available in the literature do not necessarily indicate causation of the neurologic disorders but may underline the neuropsychiatric impact associated with COVID-19.

## Plausible Neurotropic and Neuroinvasive Mechanisms

The understanding of previously known coronaviruses and the SARS-CoV-2 offers clues regarding the neurotropic and neuroinvasive potential of these viruses in humans. Upon host-infection, coronavirus may target the nervous system by causing inflammation followed by demyelination. Interestingly, a previous study also suggested the existence of hypothetical ‘brain-lung-brain axis’ as lung injury has been demonstrated to be associated with brain damage and neurocognitive dysfunction, and vice versa (Stevens and Puybasset, 2011). Evidence suggest that SARS-CoV-2 may invade the brain, particularly of immune-compromised population, through either direct or indirect routes. Other human coronaviruses (HCoVs) have previously been observed in the brain tissues by autopsy studies. HCoV strains OC43 and 229E were determined in 44 brain donors (out of 90) by the RT-PCR (Edwards et al., 2000). Particularly, the OC43 strain was found in a higher amounts in patients with multiple sclerosis as compared to the controls. A similar study also demonstrated an over-expression of MCP-1 chemokine mRNA in the astrocytes following OC43 infection (Stamatovic et al., 2005). Interestingly, elevated MCP-1 expression has been implicated in the enhanced blood–brain barrier (BBB) permeability (Glass et al., 2004). Thus, it is evident that there is a higher association of multiple sclerosis with HCoV infection. Moreover, it also implies that coronavirus infection may contribute to the pre- or co-existing neuropathology to cause chronic neurologic complications.

**FIGURE 2:**
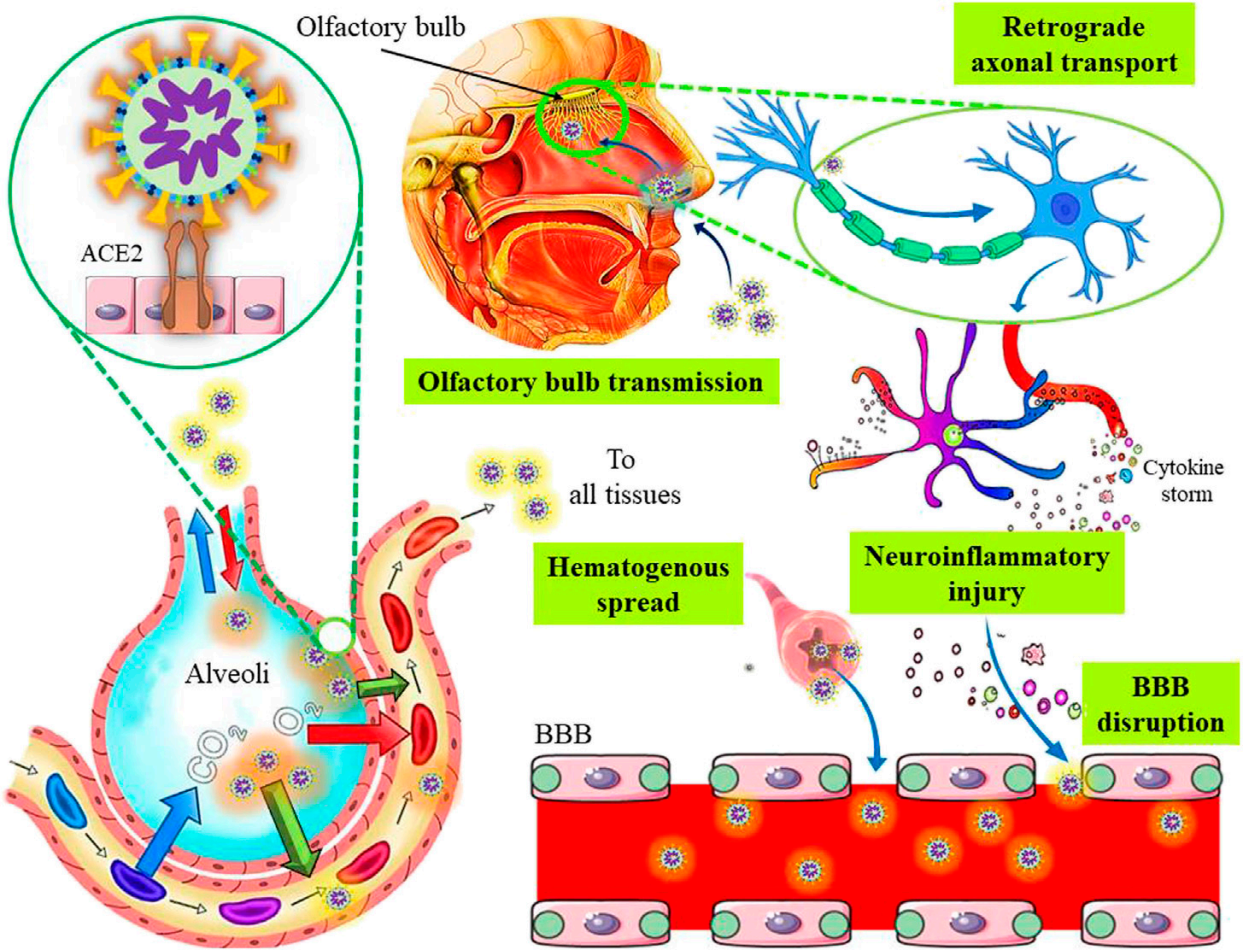
The plausible neurotropic and neuroinvasive mechanisms of severe acute respiratory syndrome coronavirus 2.

As described in the previous section, spike proteins of SARS-CoV-2 bind with the ACE2 receptors on the host cells, and enter inside the cell either by membrane fusion or endocytosis. The ACE2 receptors are also expressed in neurons of different brain regions, which can bind to the integrins and regulate integrin signaling (Doobay et al., 2007; Clarke et al., 2012). Recently, an integrin-binding motif in the S protein of SARS-CoV-2 was recognized, suggesting that an ACE2-independent cell invasion might be possible in integrin-expressing cells (Sigrist et al., 2020). The neurotropic nature of SARS-CoV-2 was confirmed by the presence of the virus in the CSF of a COVID-19 patient with viral encephalitis (Moriguchi et al., 2020) as well as in the brain of a deceased COVID-19 subject with resting tremors and gait impairment due to Parkinson's disease (Paniz-Mondolfi et al., 2020). The presence of SARS-CoV-2 was also reported in the olfactory epithelium, olfactory bulbs, trigeminal ganglia, brainstem, uvula, cornea, and conjunctiva of some corpses (Meinhardt et al., 2021). A hypothesis suggests the nerve endings in the conjunctiva and oral/nasal mucosa including the olfactory nerves might act as potential entry sites for the SARS-CoV-2 infection (Cheema et al., 2020; Colavita et al., 2020). Post-mortem MRI findings of four COVID-19 positive cadavers showed asymmetric olfactory bulbs, that point towards olfactory neuroepithelium as a probable site for the virus entry (Coolen et al., 2020). The ACE2 and TMPRSS2 are also expressed in the neuroepithelium of the olfactory bulb (Fodoulian et al., 2020), which are probably associated with hyposmia and dysgeusia in COVID-19 patients during the infection. Additionally, the SARS-CoV-2 is also assumed to spread to the brainstem via olfactory bulb (direct route) or orofacial sensory fibers (alternate route) via cranial ganglia. The direct olfactory route involves the dissemination of SARS-CoV-2 to the amygdala and piriform cortex through the medial forebrain bundle that projects caudally to the dorsal vagal nuclei and solitary tract (Fenrich et al., 2020). Importantly, the COVID-19 associated dysgeusia may be explained by the viral replication in the solitary tract neurons. It is also suggested that SARS-CoV-2 can disrupt the chemoreceptors by invading the olfactory mucosa to trigger an inflammatory response (Fiani et al., 2020). The COVID-19 associated early anosmia may be a result of the early neuroinvasion, probably through the olfactory bulb as HCoV utilizes retrograde transport to reach the olfactory nerve. Experimental data suggest the presence of viral-specific antigens in the olfactory bulbs of transgenic mice following 3 days of intranasal HCoV-OC43 inoculation. Moreover, after 7 days of inoculation, viral dissemination was observed throughout the brain coinciding with clinical encephalitis. Experimental nasal inoculation demonstrated about an 8-fold increase in the SARS-CoV-positive cell density in the CNS, particularly in the hippocampus, 1–2 weeks after the infection (Chan et al., 2020). On the other hand, the alternate route of propagation through orofacial nerves also represents a plausible site for the persistent infection as well as replication, since pseudounipolar somata mainly reside in the cranial ganglia. Furthermore, it facilitates brainstem invasion either by axonal transport or exocytosis-endocytosis mediated transfection of other fibers passing through the ganglia. Nevertheless, vesicular transport may also prevail and contribute to such transmission. Cross human tissue surveys revealed co-expression of ACE2 and TMPRSS2 cells in the nasal goblet cells, ciliated cells, and oligodendrocytes (Sardu et al., 2020). Therefore, co-expression of ACE2/TMPRSS2 proteins in the oligodendrocytes might be one route of CNS infiltration or proliferation. Although it is being hypothesized that viremia can allow the virus to reach the cerebral circulation to promote neurotropic effects, the observed discrepancy between the neurological manifestations (Mao et al., 2020) and absence of virus in the blood samples (Wölfel et al., 2020) indicates that viremia is unlikely to be a significant contributor of viral invasion to the brain, contradicting the hypothesis of hematogenous transmission of the virus in the host. Usually, the hematogenous route leads to the infection of the BBB endothelial cells and blood–cerebrospinal cells in the choroid plexus as shown in Figure 2 (Bohmwald et al., 2018). Apart from these, the induction of respiratory stress caused by lung damage can subsequently induce multi-organ failure through cascade effect and neuronal insults (Fiani et al., 2020).

The compromised BBB due to endothelial injury, inflammatory mediators, infected macrophages, or direct infection of the endothelial cells, also represent an alternate way for virus neuroinvasion (Sardu et al., 2020). Cytokine storms play an important role in both acute lung damage and neurotoxicity (Wang et al., 2010). The BBB integrity can be disrupted by cytokine- and/or immune-mediated injuries in the absence of direct viral invasion (Figure 2). A previous study suggests that acute necrotizing encephalopathy may occur due to the cytokine-mediated inflammations (Ouattara et al., 2011). Furthermore, neuroinflammatory insults leading to functional brain damage may partly explain the cognitive deficits associated with viral pneumonia. The systemic inflammation in specific cortical regions may also cause altered consciousness as well as other behavioral changes (Sasannejad et al., 2019). Although the hyper-active cytokine response observed in SARS-CoV-2 infection may impact the neurologic complications by manipulating the neuro-inflammatory pathways, the exact mechanisms involved are yet to be determined. Apart from neuro-inflammation, prolonged hypoxia may also promote cognitive impairments and neuropsychiatric sequelae (Steardo et al., 2020). Particularly, neuro-inflammation has been implicated in other neurodegenerative and psychiatric disorders such as schizophrenia and acute psychosis (Pape et al., 2019). On the other hand, stress can also activate hypothalamic-pituitary-adrenal (HPA) axis, which leads to the high production of steroids that may contribute to the impairment of the immunological functioning and exacerbation of psychological conditions (Steenblock et al., 2020).

The CNS infiltration by SARS-CoV-2 via peripheral nerves is a multi-stage process. The virus requires the exploitation of the retrograde axonal transport machinery to access the neuronal soma from the peripheral neurons (Figure 2). As evident, SARS-CoV-2 usually utilizes the ACE2-mediated endocytotic pathway for virus internalization followed by intracellular transport. The intrinsic clathrin-independent intracellular ACE2-mediated endocytosis is also implicated. Moreover, for a successful invasion, the virus must be capable of crossing the synaptic membranes. Notably, an earlier-known beta-coronavirus exhibited trans-synaptic transmission through presynaptic endocytosis as well as postsynaptic exocytosis (Li et al., 2013), which implies that SARS-CoV-2 might use a similar mechanism. In addition, viral dissemination via neurons from the ENS to the CNS is also possible by anterograde pathways, as this route usually reach the same brain sites like that of retrograde transport (Parker et al., 2020). Generally, kinesin-mediated anterograde axonal transport allows the trafficking of vesicles from the soma to the axon or axonal ends (Berth et al., 2009). As SARS-CoV-2 also forms ERGIC, it could manipulate the kinesin-mediated anterograde route to disseminate along the axons (Fenrich et al., 2020). Also, lateral transfections, either cell to- cell or axo-axonal spreading could be possible. Moreover, it was revealed that ACE2 trafficking could be involved in the exosome-dependent cell-to-cell transfer, probably assisting the infection in cerebrovascular endothelial cells (Wang J. et al., 2020).

Besides, SARS-CoV-2 may utilize peripheral nerves like the trigeminal nerve that has sensory innervation of the vagus nerve or the nociceptive cells that originate from the brain stem and supplies to several regions of the respiratory tract like the trachea, larynx, and lungs (Koyuncu et al., 2013; Meshkat et al., 2020). Previous evidence suggests the possibility of direct brainstem invasion by the viruses (particularly with pseudorabies virus) through chemoreceptors and mechanoreceptors found in the lungs as well as in the lower respiratory tract (Hadziefendic and Haxhiu, 1999), a possible way that can be adopted by SARS-CoV-2 to invade the CNS. However, it is yet to be established. Notably, the SARS-CoV-2 invasion of the spinal cord and brainstem practically allows the virus to target every organ system of the body (Alam et al., 2020). For instance, infection of vagal nuclei alone may allow viral dissemination to the heart, lungs, intestines, liver, and kidneys. As the lungs represent the initial reservoir for the virus, it is conceivable that SARS-CoV-2 could use the vagus nerve to invade the CNS via the lung–gut–brain axis (Shinu et al., 2020). This also allows the virus to potentially interfere with all the systems of axis at various time-points during the SARS-CoV-2 infection, which may justify the occurrence of a combination of respiratory, gastrointestinal, and neurological (neuropathic) symptoms in certain patients throughout the course of infection (Alam et al., 2020). This independently may lead to multiple organ failure in the absence of respiratory pathology. Therefore, vagal dysfunction might be considered a significant contributor to the amplified immune responses and thromboembolic events in some COVID-19 subjects (Niimi and Chung, 2015). Interestingly, vagal neuropathies associated with viral upper-respiratory-tract infections are already recognized clinically leading to para- and post-infectious sequelae (Niimi and Chung, 2015). Although the exact time required for viral invasion is not determined well, it is certainly dependent on the route of virus entry and viral load. Based on the axonal transport dynamics via active and passive processes, the CNS infection may develop within a week after the virus exposure (Fenrich et al., 2020).

## Neurologic Manifestations and Sequelae: The Growing Evidences

Despite an emphasis on respiratory complications, the evidence of neurological manifestations of SARS-CoV-2 infection is rapidly growing, which is substantially contributing to morbidity and mortality. During the initial wave of COVID-19 in China, a single-centre, retrospective study (n = 99) demonstrated the occurrence of confusion and headache in about 9 and 8% subjects, respectively (Chen N. et al., 2020). Following this, another retrospective, observational case series, analyzing 214 patients revealed that about 36.4% (n = 78) had neurologic manifestations, which were categorized as CNS manifestations (headache, dizziness, impaired consciousness, ataxia, acute cerebrovascular disease, and seizure), PNS manifestations (hyposmia, hypogeusia, vision impairment, and neuralgia), and skeletal muscle injury manifestations (Mao et al., 2020). Various evidence are available in the existing literature regarding the higher association of headache and dizziness following SARS-CoV-2 infection (Chen N. et al., 2020; Guan et al., 2020; Huang et al., 2020; Mao et al., 2020). Similarly, clinical reports have also demonstrated the frequent incidence of olfactory and gustatory dysfunctions in COVID-19 patients, causing hyposmia and hypogeusia, respectively (Giacomelli et al., 2020; Lechien et al., 2020a; Vaira et al., 2020). An observational study from France showed a higher percentage (84%) of neurologic complications, with agitation being the most common (69%), followed by corticospinal tract signs (67%) and dysexecutive syndrome (36%) at the discharge time (Helms et al., 2020a). Remarkably, there are growing evidence on neurological manifestations and sequelae that will be discussed in the later sub-sections, considering the available incidences of direct, indirect, or post-infective complications. Although it is quite hard to distinguish the complex neurological manifestations, we tried to categorize and discuss them briefly in the following subsections, based on their occurrence in the COVID-19 patients.

### Myalgia and Other Muscle Injuries (Occurs Commonly)

An initial retrospective study from China reported the overall occurrence of myalgia in 40% cases including both moderate (30%) and severe (50%) cases (Chen G. et al., 2020). Similar data were also reported by other studies with the prevalence of 34.8% (Wang D. et al., 2020), 35.8% (Li et al., 2020), and 44% (Huang et al., 2020), respectively. As reported by the European prospective study conducted on mild-to-moderate cases of COVID-19, about 62.5% patients had myalgia in the setting (Lechien et al., 2020b). Another prospective cohort study from New York reported a prevalence of 26% of myalgia in critically ill patients (Cummings et al., 2020). On the contrary to all the above observations, a recent pooled analysis claimed that the presence of myalgia is not statistically associated with COVID-19, thus it should not be considered as a prognostic factor in severe COVID-19 cases (Lippi et al., 2020). An observational case series suggested that muscle injury (i.e., myalgia with elevated serum creatine kinase above 200 U/L) is more common in severe cases as compared to non-severe cases (19.3 vs. 4.8%), and patients with muscle injury are comparatively at the high-risk of developing multi-organ failure, including serious kidney and liver abnormalities (Mao et al., 2020). Evidence also suggest the development of rhabdomyolysis in rare cases, as a potential late complication of SARS-CoV-2 infection (Jin and Tong, 2020). Although electromyography and muscle imaging or histopathology is not available to date, the available data suggests that SARS-CoV-2 infection may also likely cause viral myositis. Likewise, infected patients may probably develop muscle weakness due to muscular atrophy from critical illness myopathy and polyneuropathy, but specifically constructed analyses are yet to be planned (Guidon and Amato, 2020). Nevertheless, it is also plausible that various skeletal muscle types may exhibit susceptibility to SARS-CoV-2 via ACE2 receptors expressed on the muscles (Fernandes et al., 2010; Ferrandi et al., 2020), independent of the nervous system involvement. Additionally, SARS-CoV-2 infection elicits the release of cascade of cytokines, like IL-6, which can also disrupt the muscle metabolic homeostasis and exacerbate muscle loss (VanderVeen et al., 2019; Ferrandi et al., 2020). Thus, muscle injuries may directly result from viral interaction with ACE2 receptors on muscles and/or indirectly through systemic cytokine mediated disruption and subsequent homeostatic perturbation (Ferrandi et al., 2020), irrespective of neuroinvasion.

### Olfactory and Gustatory Dysfunctions (Occurs Commonly in Mild Cases)

In light of expanding anecdotal evidence, the Centers for Disease Control and Prevention (CDC) have revised the list of symptoms of COVID-19 with the addition of sudden loss of smell and taste to the triad of typical cough, fever, and difficulty in breathing (CDC, 2020). An initial cross-sectional study (n = 59) reported that about 33.9% (n = 20) patients complained at least one olfactory and gustatory disorders, while 18.6% (n = 11) were found with both (Giacomelli et al., 2020). Taste alterations were common (91%) in pre-hospitalized patients, whereas equal frequency was observed in hospitalized cases (Giacomelli et al., 2020). A multicenter study (n = 417) conducted in Europe showed higher prevalence of olfactory (n = 357, 85.6%) and gustatory (n = 342, 88.8%) dysfunctions (Lechien et al., 2020a). This finding was consistent with the previous multicentre case-control study (n = 79) revealing 80.6% (n = 25) cases of smell disorders (of which 45.2% were anosmic) and 90.3% (n = 28) cases of taste impairment (with 45.2% ageusia) (Beltrán-Corbellini et al., 2020). Among the olfactory disorders, 20.4 (n = 73) and 79.6% (n = 284) cases were hyposmic and anosmic respectively. Notably, of the 76 patients (18.2%) without rhinorrhea or nasal obstruction, 79.7% were either hyposmic or anosmic, which implies that inflammation and obstruction of the nasal mucosa are not the only underlying cause of smell dysfunction. On the other hand, the gustatory dysfunctions represented either a reduction/discontinuation of taste (78.9%) or distorted taste ability (21.1%) towards different flavours. Following this, another study reported a prevalence of 64.4% among 202 enrolled patients (Spinato et al., 2020). Boscolo-Rizzo et al. have also provided the first insight into the anosmia and hypogeusia associated with mild cases of COVID-19 in Italy (Boscolo-Rizzo et al., 2020). A similar survey with 204 COVID-19 patients using Italian Sino-Nasal Outcome Test 22 (I-SNOT-22) demonstrated taste reduction in 55.4% and smell impairment in 41.7% of the subjects with only 7.8% cases of nasal obstruction (Mercante et al., 2020). Although follow-up of these aforementioned studies provides promising evidence of self-recovery subjective to smell and taste impairment without the aid of any medical intervention, certain populations may be likely presented for further treatment of unresolved symptoms. Interestingly, the olfactory and gustatory dysfunctions associated with SARS-CoV-2 infection are often the first apparent symptom and mostly atypical to the other viral infections i.e., without rhinorrhoea or nasal obstruction (Spinato et al., 2020).

### Headache (Occurs Commonly in Mild Cases)

Headache is one of the common symptoms associated with viral infections alongside fever. A meta-analysis of 59,254 cases reported headache as the fifth most common symptoms (n = 3,598, 12%) in COVID-19 patients, after fever, cough, myalgia, and dyspnea (Borges do Nascimento et al., 2020). Another retrospective, observational studies from China reported a similar incidence (n = 28 out of 214, 13.1%) of headache (Mao et al., 2020). On the other hand, a higher prevalence (70.3%) was observed in a European epidemiological study conducted in 1,420 mild to moderate patients (Lechien et al., 2020b). Therefore, this large variation in the data among the Asians and Europeans require more careful surveillance of the epidemiological impact. Notably, a report from a Spanish neurologist who himself suffered from COVID-19, described three different forms of headache as experienced in his clinical case (Belvis, 2020). Being a headache expert, he concluded that even though several types of headache appear (may be associated with cytokine storm) during the SARS-CoV-2 infection, but it seems to be underestimated due to the overemphasis on severe respiratory problems. Interestingly, correspondence from Japan has raised the concern on the headache as a probable manifestation of encephalitis or other viral meningitis, which subsequently express itself in the form of seizures and drowsiness (Moriguchi et al., 2020). Particularly, a 24-year-old young patient with no travel history presented progressive headache, fatigue, and seizures as general symptoms of paranasal sinusitis, encephalitis, post-convulsive encephalitis, and hippocampal sclerosis as determined by brain MRI. Although his RT-PCR test was negative for a nasopharyngeal swab, a CSF sample tested positive for SARS-CoV-2 RNA indicating the possible viral neuroinvasion (Moriguchi et al., 2020). Therefore, attention is required to determine the pathogenesis underpinning the headache-like symptoms, though they may appear to be simple symptoms in the COVID-19. Moreover, careful pain management should be practiced as there is no recommended specific treatment for such cases.

### Delirium and Impaired Consciousness (Occurs Commonly in Severe Cases)

COVID-19-related delirium and impairment of consciousness are probably due to septic-associated encephalopathy, probably caused by systemic inflammatory response syndrome. A study reported agitation (likely a hyperkinetic delirium) in 69% patients (n = 40/58) on the withdrawal of neuromuscular blockers. Subsequently, about 36% patients exhibited dysexecutive syndrome- exhibiting disorientation, attention deficit, and impaired movement at the time of discharge (Helms et al., 2020a). The COVID-19 and Frailty (CO-FRAIL) study described delirium to be associated more likely with the duration of hospital stay, ICU admission, and the use of ventilators. As per the study, 234 patients (33%) showed delirium, of which 12% subjects were having the pre-hospitalized conditions. Subsequently, about 55% subjects with delirium (compared to 30% patients without delirium) have died after hospitalization (Garcez et al., 2020). Another study from Italy reported delirium-onset COVID-19 in 36.8% patients, mostly with multiple comorbidities and advanced age (Poloni et al., 2020). A bicentric cohort analysis also revealed a very high prevalence (84.3%) of delirium in ICU subjects (Helms et al., 2020b). An epidemiological study showed the development of delirium in 11% patients, who also showed a higher prevalence of epilepsy and dementia (Ticinesi et al., 2020). The COVID-19-associated delirium should not be considered differently than the delirium due to other causes, and the implementation of appropriate delirium prevention and management measures at the bedside must be a deliberate priority during the pandemic. Mao *et al.* reported the presence of impaired consciousness in 14.8% subjects with severe COVID-19 as compared to the non-severe cases (2.4%) (Mao et al., 2020). Interesting findings by Chen *et al.* revealed that altered consciousness was comparatively more frequent in the deceased patients (22%) than those who eventually recovered (1%) from COVID-19. However, a distinct definition of the term altered consciousness in the setting was not stipulated (Chen T. et al., 2020).

### Ischemic Stroke (Occurs Rarely)

The patients with severe COVID-19 demonstrated a higher D-dimer level that suggests an altered state of the coagulation system (Mao et al., 2020). A retrospective study with ICU admitted COVID-19 patients (n = 184) revealed about 31% incidence of thrombotic complications including acute pulmonary embolism, ischemic stroke, deep-vein thrombosis, and systemic arterial embolism (Klok et al., 2020). An Italian study reported the occurrence of ischemic stroke in 2.5% cases, which was the primary reason for requiring hospitalization (Lodigiani et al., 2020). A similar study from the US reported only 1.1% cases of acute ischemic stroke in hospitalized COVID-19 patients (Jain et al., 2020). During the past months, several case studies of ischemic stroke in COVID-19 subjects have been reported (Barrios-López et al., 2020; Gunasekaran et al., 2020; Moshayedi et al., 2020; Tunç et al., 2020; Viguier et al., 2020). Notably, some of these cases may represent a causal link, and therefore, specifically designed studies will be highly appreciated in such cases.

### Inflammatory Neuropathies (Occurs Rarely)

Evidence are emerging for typical acute inflammatory polyneuropathies associated with the COVID-19 pandemic. For instance, a case of fulminant polyradiculoneuritis representing the Guillain-Barré-Syndrome (GBS) with locked-in syndrome in a COVID-19 patient was recently reported (Pfefferkorn et al., 2020). Previously, the first report (self-claimed) of GBS was described in a COVID-19 patient exhibiting acute progressive ascending symmetric quadriparesis as a symptom (Sedaghat and Karimi, 2020). Another report described two rare cases of polyneuritis cranialis and Miller Fisher syndrome associated with SARS-CoV-2 infection (Gutiérrez-Ortiz et al., 2020). The treatment of the subjects included acetaminophen and IV immunoglobulin (IVIg), and complete recovery of both the patients was observed within 2 weeks of the treatment. Similarly, the cranial neuropathies i.e., diplopia and ophthalmoparesis in two COVID-19 patients having abnormal perineural and cranial nerve palsy were also reported (Dinkin et al., 2020). A case series also described GBS in five patients following the onset of COVID-19 (Toscano et al., 2020). Very recently, clinicoradiologic evaluation, diagnosis, clinical progression, and multidisciplinary management of a COVID-19 patient with a recognized GBS subtype, bifacial weakness with paresthesia is also reported (Hutchins et al., 2020). Notably, a recent case study highlighted the association of GBS in an 11-year child with severe COVID-19 condition (Khalifa et al., 2020). Future studies are expected to determine the clinical as well as electrophysiological characteristics of COVID-19-associated GBS and its variant along with the establishment of their causal relationship. However, the treatment of para- or post-COVID-19 GBS is similar to that of other inflammatory neuropathies.

### Other Rarer Neurologic Manifestations

Apart from the above discussed neurologic complications, some of the rare cases of neurologic manifestations were also reported, including cerebral venous thrombosis (Hemasian and Ansari, 2020; Hughes et al., 2020; Poillon et al., 2020), intracerebral hemorrhage (Al-olama et al., 2020; Carroll and Lewis, 2020; Sharifi-Razavi et al., 2020), status epilepticus (Balloy et al., 2020; Somani et al., 2020; Vollono et al., 2020), generalized myoclonus (Rábano-Suárez et al., 2020), seizures (Anand et al., 2020; García-Howard et al., 2020), acute epileptic encephalopathy (Mahammedi et al., 2020), hemorrhagic posterior reversible encephalopathy syndrome (Franceschi et al., 2020), acute necrotizing encephalopathy (Dixon et al., 2020), steroid-responsive encephalitis (Pilotto et al., 2020), diffuse leukoencephalopathy (Radmanesh et al., 2020; Sachs et al., 2020), neuroleptic malignant syndrome (Kajani et al., 2020), and post-infectious transverse myelitis (Munz et al., 2020). Similar evidence are growing on the association of meningoencephalitis with COVID-19 (Chaumont et al., 2020; Dogan et al., 2020; Duong et al., 2020; Mardani et al., 2020). Concerns have been raised on the development of multiple sclerosis in COVID-19 patients as well (Bowen et al., 2020; Louapre et al., 2020). However, the full scope of COVID-19 complications in multiple sclerosis patients remains to be defined. Among all these complications, seizures are observed quite commonly in the elder patients; but it may not be directly related to the SARS-CoV-2 infection. It has been suggested that patients with critical COVID-19 exhibiting mental complications must be subjected to continuous EEG monitoring for the possible occurrence of nonconvulsive status epilepticus (Asadi-Pooya and Simani, 2020). Although numerous cases have been reported on COVID-19 associated seizures/epilepsy in the past months, it is of utmost importance to validate the possible drug-drug interactions between antiseizure drugs (particularly phenytoin, phenobarbital, carbamazepine, and primidone) and the drugs used for the treatment of COVID-19 (Orsucci et al., 2020). However, more epidemiological data are required to establish a direct causal relationship between COVID-19 and the above-mentioned rarer neurological characteristics.

### Psychological Impact

The advent of the pandemic surged distress around illness, mortality, and uncertainty about the future amidst the general public and COVID-19 patients along with a consequential alteration in psychosocial behavior. Additionally, lockdown implementation, loss of organized educational framework, high unemployment rate, and social distancing further contribute towards the increase in the detrimental mental issues (Kola, 2020; Moreno et al., 2020; Pfefferbaum and North, 2020). Mental illness itself possesses the greatest threat to the daily habits, lifestyle, socioeconomic status as well as mortality and morbidity associated with COVID-19 (Walker et al., 2015), which also affects the clinical outcomes. Furthermore, altered psychology can cause abnormal perception and thinking, impaired social behavior, delusions, hallucinations, cognitive dysfunction, and social isolation that may result in poor treatment adherence, and non-seeking of health care facilities. Patients with a serious mental illness ultimately live a compromised quality of life (Evans et al., 2007). Patients with a pre-existing severe mental illness shown to have 2-3 folds higher risk for severe clinical outcomes as compared to patients with no history of mental disabilities (Lee et al., 2020). A cross-sectional study reported the psychological impact of SARS-CoV-2 infection in Spain. It revealed that about 21.6, 18.7, and 15.8% patients were diagnosed with anxiety, depression, and post-traumatic stress disorder (PTSD), respectively. In addition to that personal economic condition, retirement, and age factor also contributed to the progression of anxiety, depression, and PTSD (González-Sanguino et al., 2020). A meta-analysis showed the prevalence of anxiety (23.2%) and depression (22.8%) with variability among males and females (Pappa et al., 2020). Another study disclosed a higher psychological impact on younger people and comorbid patients (Ozamiz-Etxebarria et al., 2020). Moreover, unjustified fear of COVID-19 leads to elevated anxiety among the general population and in comorbid patients leading to stigmatization and discrimination (Mowbray, 2020). Bao *et al.* suggested the development of mental health screening programs as well as the implementation of such interventions for both the healthcare workers and the public (Bao et al., 2020). Notably, the COVID-19 outbreak has brought new ventures in psychological treatment as the interface of COVID-19 and psychiatry is relevant to infected and non-infected patients. Even though lockdown instigation led to the teleconsultation, there is a necessity for proper close-up monitoring and management of the medication-related adverse events.

## Neurologic Implications Associated With Off-Label Use of Drugs Against COVID-19

To date, no effective therapeutic interventions have been approved for the SARS-CoV-2 infection. As per the Infectious Diseases Society of America (IDSA) Guidelines on the Treatment and Management of Patients with COVID-19, interventions containing chloroquine (CQ), hydroxychloroquine (HCQ), teicoplanin, ivermectin, tocilizumab, lopinavir/ritonavir (LPV-r) combination, and convalescent plasma therapy are to be considered only in the clinical trial settings. Similarly, drugs like remdesivir, favipiravir, and corticosteroids are undergoing investigations for COVID-19 management, particularly in hospitalized or critically ill patients. Some Chinese guidelines also recommended the use of umifenovir (Arbidol), intravenous immune globulin (IVIG), and nebulized interferon-α in the COVID-19 treatment (Dong et al., 2020; Jin Y.-H. et al., 2020). Importantly, some of these experimental drugs have been reported to possess certain neurologic adverse drug reactions (ADRs), which are discussed below (Table 1).

**TABLE 1:** The potential mechanism(s) and neurologic adverse effects of the COVID-19 therapies.

Drug	Mechanism(s)	Neurologic adverse effects
CQ	Alteration of acidification of endosomes to interfere with the cellular functions, interfere with the binding of virus to the ACE2 receptor, inhibition of cytokine effect	Psychosis, anxiety, agitation, irritable or blunted mood, seizure, bipolar mood disorder, delirium, reversible vacuolar myopathy, extrapyramidal disorders (Parkinsonism, dystonias and oculogyric crisis), ototoxicity
HCQ		Ataxia, loss of hearing, vertigo, dizziness, tinnitus, psychosis, reversible vacuolar myopathy, seizure
Azithromycin	No direct mechanisms. Given as adjunct to CQ/HCQ against community-acquired pneumonia (CAP)	Headache, dizziness, vertigo, catatonia, psychotic depression, delirium, anxiety, somnolence
Lopinavir/Ritonavir (LPV-r)	Inhibition of protease	agitation, abnormal dreams, confusion, anxiety, emotional disturbances, neurotoxicity, paresthesias, taste alterations
Tocilizumab	IL-6 inhibition	Headache, dizziness, peripheral neuropathy, leukoencephalopathy, cognitive impairment, demyelinating disorders, depression
Corticosteroids	Modulation of hyper-inflammatory state and regulation of immune responses	Agitation, anxiety, depression, delusion, hallucinations, seizure, acute steroid myopathy, myalgia
Interferon α	Modulation of immune responses	Anxiety disorders, fatigue, apathy, irritability, mood disorders, cognitive deficits, suicidal tendency, sleep disturbances
Umifenovir	Interfere with the clathrin-mediated endocytosis	Dizziness, acute psychiatric symptoms
Favipiravir	Selective inhibition of RNA-dependent RNA polymerase (RdRP)	Psychiatric reactions

CQ, Chloroquine; HCQ, Hydroxychloroquine. The possible drug-drug interactions can be checked at https://www.covid19-druginteractions.org.

Chloroquine and Hydroxychloroquine were both originally developed as antimalarial agents that act by averting the acidification of endosomes to interfere with the cellular functions and/or interfere with the binding of the virus to the ACE2 receptor (Borah et al., 2020). Although *in vitro* studies suggest the potential of HCQ against SARS-CoV-2, but *in vivo* data are still lacking. Apart from the cardiovascular side effects, several other reports also suggest the possible adverse neurologic reactions associated with the CQ/HCQ usage. For instance, the CQ-induced psychosis was first observed in 1958 (Burrell and Martinez, 1958). CQ administration also exhibited the induction of seizures in some patients. These CQ-induced seizures are suggested to be a possible idiosyncratic reaction (Luijckx et al., 1992; Krzeminski et al., 2018). Similarly, HCQ can also lower the seizure threshold, and thus interact with certain antiepileptic drugs like lacosamide and lamotrigine (Fish and Espir, 1988). Extrapyramidal disorders like Parkinsonism, dystonias, and oculogyric crisis were also demonstrated to be associated with CQ/HCQ administration (Parmar et al., 2000; Busari et al., 2013). Previous studies also reported the CQ-induced (dose-independent) psychotic features like anxiety, agitation, irritable or blunted mood, and bipolar mood disorder, accompanied by hallucination, derealization, and positive symptoms (Biswas et al., 2014). Reversible vacuolar myopathy is the common type of myopathy associated with the use of CQ/HCQ. Moreover, the CQ/HCQ-induced myopathy is further contributed by factors like Caucasian ethnicity, renal failure, connective tissue disorders, long-term corticosteroid therapy, and co-administration of proton pump inhibitors, statins, and myotoxic agents (Khosa et al., 2018). Additionally, dose-dependent retinopathy and maculopathy are also related to CQ administration, particularly in elder patients. A daily dose comprising more than 4 mg/kg/day may precipitate such conditions (Elman et al., 1976). HCQ is well known to aggravate the myasthenia gravis and therefore, contraindicated in those patients. Interestingly, CQ/HCQ-induced ototoxicity like loss of hearing, vertigo, tinnitus, and disequilibrium, may mimic stroke-like condition in COVID-19 patients (Hadi et al., 1996; Khalili et al., 2014). Due to the prompted systemic adverse events, a novel non-systemic low-dose aerosol formulation with 2–4 mg/inhalation dose has been suggested to minimize the ADRs related to CQ/HCQ usage (Klimke et al., 2020). Several mechanisms have been proposed for the pathogenesis of HCQ-induced neuropsychiatric events, such as inhibition of serotonin transporter, N-methyl-D-aspartate (NMDA) antagonism, acetylcholinesterase inhibition, and gamma aminobutyric acid (GABA) antagonism (Good and Shader, 1977). The metabolism of both CQ and HCQ is mainly done by the CYP3A4 enzyme, therefore, CYP3A4 inhibitors like fluvoxamine could raise the plasma levels and further potentiate the adverse effects. On the other hand, CYP3A4 inducers like oxcarbazepine, carbamazepine, and modafinil, could reduce the plasma levels of CQ and HCQ and render them less effective. As the half-life of HCQ is comparatively longer (40 h), the potential adverse effects may last for days after the discontinuation of the drug (Browning, 2014). Azithromycin, an antibacterial drug has been investigated in conjunction with CQ or HCQ in several clinical trial settings. This drug is also known to precipitate certain neuropsychiatric events like headache, dizziness, vertigo, catatonia, psychotic depression, delirium, anxiety, and somnolence (Ginsberg, 2006). Interestingly, CQ, HCQ and azithromycin, all can interfere with the heart conduction system and may lead to prolonged QT interval, blockade of bundle conduction, atrioventricular blockade, and torsades de pointes (Leitner et al., 2010; McGhie et al., 2018). Therefore, caution should be practiced while combining these drugs with psychotropic agents that affect the QT interval.

LPV-r co-formulation is used in the treatment of HIV-1 infection. The co-administration of ritonavir enhances the half-life of lopinavir (mainly a protease inhibitor) by inhibiting CYP450 metabolism (Borah et al., 2020). A Korean study showed viral load reduction in a COVID-19 patient with LPV-r therapy (Lim et al., 2020), but another randomized, open-label trial demonstrated no significant therapeutic benefit of it (Cao et al., 2020). Although there is limited data on neuropsychiatric adverse events, the manufacturer suggests the possible effects like agitation, abnormal dreams, confusion, anxiety, and emotional disturbances associated with the drug (FDA, 2016). Moreover, protease inhibitors are well known to cause neurological adverse effects, such as neurotoxicity, paresthesias, and taste alterations (Abers et al., 2014). Notably, protease inhibitors are highly metabolized by the cytochrome P450 enzymes, and therefore, demonstrate drug-drug interaction with many drugs, including the psychotropic agents, which are major substrates for the CYP isoenzymes (Goodlet et al., 2019). The use of LPV-r is contraindicated with drugs like midazolam, triazolam, and pimozide, because it may potentiate the adverse effects by enhancing the concentrations of co-administered drugs. Thus, the use of benzodiazepines like lorazepam, temazepam, or oxazepam, which are independent of CYP metabolism, is recommended in such cases. On the contrary, ritonavir-co-formulated protease inhibitors also lower the drug concentration of few psychotropic agents including bupropion, lamotrigine, methadone, and olanzapine, due to glucuronidation effects or CYP metabolism (Goodlet et al., 2019). Therefore, the clinicians should carefully assess the potential drug-drug interactions to prevent unintentional adverse effects.

Tocilizumab is a monoclonal antibody approved for the treatment of rheumatoid disorders and chimeric antigen receptor-T cell (CAR-T)-mediated CRS (NIH, 2020). Based on the preliminary evidence, China recommended the use of tocilizumab in severe or critical COVID-19 cases (National Health Commission and State Administration of and Traditional Chinese Medicine, 2020). However, the National Institutes of Health (NIH) Panel recommended against the tocilizumab usage stating that there is no adequate data regarding its efficacy (NIH, 2020). Tocilizumab demonstrated very poor CNS penetrating ability (Nellan et al., 2018). Although no severe neurologic ADRs have been reported with tocilizumab usage, but headache, dizziness, peripheral neuropathy, hypertension, leukoencephalopathy, cognitive impairment, gastrointestinal perforations, hypersensitivity reactions, including anaphylaxis and demyelinating disorders may occur in certain patients (Tanaka et al., 2010; Sheppard et al., 2017; Farooqi et al., 2020). Study conducted on patients with rheumatoid arthritis exhibited the tocilizumab-associated depressive symptoms (Singh et al., 2011; Harrold et al., 2017). Moreover, cases of multifocal cerebral thrombotic microangiopathy are also rarely observed (Jewell et al., 2016).

Corticosteroids are also known to modulate hyper-inflammatory state and regulate immune responses that are necessary for the host defense mechanisms. The corticosteroid administration in COVID-19 patients is recommended on a case-by-case basis based on drug indications, illness severity, and comorbid conditions (Borah et al., 2020). Although strong recommendation on the routine use of systemic corticosteroids in mechanically ventilated COVID-19 patients without ARDS is provided, the use of systemic corticosteroids in the treatment of hospitalized COVID-19 patients is not advised (WHO, 2020a). On the other hand, low-dose corticosteroid therapy is prescribed in adult COVID-19 patients with refractory shock (NIH, 2020). However, several corticosteroid-associated neurologic ADRs have been previously reported. Corticosteroid administration could induce mood disorders including agitation, anxiety, and depression (Ou et al., 2018). Therefore, clinical pharmacists and neurologists should be aware of such ADRs in COVID-19 patients with ARDS, particularly those with a history of stroke treated with corticosteroids. Similarly, corticosteroids may also induce dose-dependent psychiatric illness like delusion and hallucinations in older patients with a stroke experience (Patten and Neutel, 2000; Wada et al., 2001; Hodgins et al., 2018). In addition to these, corticosteroid therapy may rarely trigger the convulsions by acting on mineralocorticoid receptors (Jaenisch et al., 2016). Prolonged high-dose steroid therapy could also present acute steroid myopathy and myalgia (Sun and Chu, 2017). Since these adverse reactions may occur in SARS-CoV-2 infection, close monitoring of the patients during corticosteroid therapy is an important pre-requisite.

Interferons (IFNs) are glycoproteins having potential immunomodulatory and hormone-like functions (Jacobs and Johnson, 1994). Both IFNα and IFNβ have been considered as a potential therapy against COVID-19, particularly in combination with ribavirin (Lu et al., 2020). Notably, IFNα comes with a boxed warning stating “life-threatening or fatal neuropsychiatric disorders” (FDA, 2017). This represents particular events such as anxiety disorders, fatigue, apathy, irritability, mood disorders, cognitive deficits, suicidal tendency, and sleep disturbances (Davoodi et al., 2018). On the other hand, neuropsychiatric adverse effects of IFNβ include fatigue and myalgia (Reder and Feng, 2014). Considering the significant neuropsychiatric adverse effects of IFNα, the clinicians should scrutinize the psychiatric history of the patient followed by close monitoring for the emergence of such symptoms. It is suggested that the concurrent use of psychotropic agents such as carbamazepine, clozapine, and valproate should be carefully evaluated for bone marrow suppression. Apart from this, the use of bupropion in conjunction has been implicated in the initiation of seizures (Ahmed et al., 2011).

Few other drugs such as remdesivir, umifenovir, and favipiravir are also undergoing clinical investigations. However, very little is known about the potential neurologic side effects associated with these drugs. Remdesivir is a comparatively well-tolerated drug with less serious adverse reactions. Umifenovir (Arbidol) is known to induce dizziness and acute psychiatric symptoms but is generally considered as a safe and well-tolerated agent. Similarly, favipiravir might also rarely provoke drug-associated neuropsychological complications (Ghasemiyeh et al., 2020). Most importantly, the potential drug-drug interactions must be considered during the concomitant use of other agents used for the management of the coexisting neuropsychiatric conditions. For detailed information, the guidance on potential drug-drug interactions can be found on the website of the University of Liverpool (https://www.covid19-druginteractions.org.)

## Conclusion

Although considerable surveillance data have been gathered regarding the direct respiratory damage caused by SARS-CoV-2, the emerging evidence revealed the involvement of the nervous system in the pathogenesis of the COVID-19. The COVID-19-associated neurological manifestations may range from mild symptoms, such as dizziness and headache to severe complications like stroke and encephalitis. Hypothetically, SARS-CoV-2 may affect CNS either by direct mechanisms like neuronal retrograde and hematogenous dissemination or via indirect pathways. However, the patients with neuroinvasion in the early stages of infection may remain unidentified and misdiagnosed, which may further contribute to the inadvertent spread of the virus. Though the precise neuropsychiatric burden of SARS-CoV-2 infection is yet to be deciphered, it is expected to have a substantial impact for several years to come. Taking the present scenario of SARS-CoV-2 infection and casualties into consideration, it is imperative to elucidate the neurologic involvement in the disease progression. The brain autopsy may be considered as a valuable facet in identifying the potential neuroinvasive mechanisms of the virus. The limited data available in the literature on the COVID-19-associated neuropsychiatric manifestations and sequelae also indicates the under-reporting of such cases in the setting of co-existing predominant cardiopulmonary complications, which make it quite difficult to accomplish comprehensive neurological investigations, especially in severe COVID-19 cases where the concurrence of such complications may be more common. Furthermore, severe or critically ill COVID-19 patients are kept under strict isolation, where obtaining neuroimaging data is limited or restricted. Thus, a close follow-up of the subjects remains constrained. Nevertheless, continuous efforts should be made to tackle these hurdles to better illustrate the pathogenesis of SARS-CoV-2, and its neurotropic as well as neuroinvasive potential. Optimistically, these findings will help the clinicians to identify plausible neurobiological targets and detect the early signs of neuropsychiatric complications to prompt therapeutic interventions before the irreversible neurologic injury. Moreover, a systematic neurological follow-up of the recovered patients may warrant a better understanding of the neurological sequelae of the viral infection. Most importantly, emphasis should be given to creating awareness among the general public to reduce the negative social attitude and extreme fear associated with the COVID-19 pandemic, which will undoubtedly improve the social and mental well-being of the people.

While the entire world eagerly awaits a potent and effective prophylactic intervention, the current management approaches are mainly focused on drug-based treatments. The therapeutic potential of certain repurposed drugs has led to their off-label use against COVID-19. Unfortunately, the associated clinical neuropsychiatric adverse events of some of these drugs remain a critical issue. Moreover, patients prescribed with these treatments are often hospitalized or seriously ill, and also receiving concomitant medications. Thus, these potential COVID-19 drugs may also interact with the concomitant medications prescribed for pre-existing or concurrent neuropsychiatric diseases, thereby, further complicating the condition. Therefore, COVID-19 represents a major threat to the field of neuropsychiatry, as both the virus and the potential therapies may induce neurologic as well as psychiatric disorders. Keeping this in mind, the neuropsychologists must be accustomed to the neuropsychiatric consequences of SARS-CoV-2 as well as the prescribed drugs, and potential drug-drug interactions of the concomitant medications. Particularly, the neuropsychologist treating COVID-19 patients should review all the medications and monitor the possible neuropsychiatric adverse events related to the medications such as HCQ or corticosteroids, to differentiate the primary and secondary (drug-induced) psychiatric complications.

## Abbreviations

ACE2, angiotensin-converting enzyme 2; ADR, adverse drug reaction; ARDS, acute respiratory distress syndrome; BBB, blood–brain barrier; CDC, centers for disease control and prevention; CNS, central nervous system; COVID-19, coronavirus disease-2019; CQ, chloroquine; CRS, cytokine release syndrome; CYP, cytochrome P; ERGIC, endoplasmic reticulum-golgi intermediate compartment; GBS, Guillain-Barré-syndrome; hCoVs, human coronaviruses; HCQ, hydroxychloroquine; IDSA, infectious diseases society of America; IFN, interferon; IL-6, interleukin-6; IVIg, intravenous immunoglobulin; LPV-r, lopinavir/ritonavir; MCP1, monocyte chemo-attractant protein 1; PTSD, post-traumatic stress disorder; RBD, receptor binding-domain; RdRP, RNA-dependent RNA polymerase; RTC, replication-transcription complex; SARS-CoV-2, severe acute respiratory syndrome coronavirus 2; TMPRSS2, transmembrane protease serine 2.
